# *Francisella philomiragia* bacteremia in an immunocompromised patient: a rare case report

**DOI:** 10.1186/s12941-021-00475-2

**Published:** 2021-10-03

**Authors:** Hui Shan Chua, Yih Harng Soh, Shih Keng Loong, Sazaly AbuBakar

**Affiliations:** 1 Department of Pathology, Hospital Melaka, Jalan Mufti Haji Khalil, 75400 Melaka, Malaysia; 2 Unit of Infection Control, Hospital Melaka, Jalan Mufti Haji Khalil, 75400 Melaka, Malaysia; 3 Tropical Infectious Diseases Research & Education Centre (TIDREC), High Impact Research Building, Universiti Malaya, 50603 Kuala Lumpur, Malaysia; 4 Department of Social and Preventive Medicine, Faculty of Medicine, University of Malaya, 50603 Kuala Lumpur, Malaysia; 5 Department of Medical Microbiology, Faculty of Medicine, University of Malaya, 50603 Kuala Lumpur, Malaysia

**Keywords:** 16S rDNA, *Francisella philomiragia*, Infectious disease, Malaysia, MALDI-TOF, Pneumonia

## Abstract

**Background:**

*Francisella philomiragia* is a very rare opportunistic pathogen of humans which causes protean diseases such as pneumonia and other systemic infections. Subsequent failure of prompt treatment may result in poor prognosis with mortality among infected patients.

**Case presentation:**

The present report describes a case of *F. philomiragia* bacteraemia first reported in Malaysia and Asian in a 60-year-old patient with underlying end-stage renal disease (ESRF) and diabetes mellitus. He presented with Acute Pulmonary Oedema with Non-ST-Elevation Myocardial Infarction (NSTEMI) in our hospital. He was intubated in view of persistent type I respiratory failure and persistent desaturation despite post haemodialysis. Blood investigation indicated the presence of ongoing infection and inflammation. The aerobic blood culture growth of *F. philomiragia* was identified using the matrix-assisted laser desorption/ionization-time of flight (MALDI-TOF) mass spectrometry (Score value: 2.16) and confirmed by 16S Ribosomal DNA (16S rDNA) sequencing. He was discharged well on day 26 of admission, after completing one week of piperacillin/tazobactam and two weeks of doxycycline.

**Conclusion:**

Clinical suspicion should be raised if patients with known risk factors are presenting with pneumonia or pulmonary nodules especially as these are the most common manifestations of *F. philomiragia* infection. Early diagnosis via accurate laboratory identification of the organism through MALDI-TOF mass spectrometry and molecular technique such as 16S rDNA sequencing are vital for prompt treatment that results in better outcomes for the afflicted patients.

## Background

*Francisella philomiragia*, previously known as *Yersinia philomiragia*, belonging to the family *Francisellaceae*, is a strictly aerobic, Gram-negative coccobacillus [1, 2]. In contrast to its more virulent relative *Francisella tularensis, F. philomiragia* is a rare opportunistic pathogen of humans which causes protean diseases such as pneumonia and other systemic infections in immunocompromised patients and near-drowning victims [1]. Limited knowledge and the lack of awareness of this organism have led to the misidentification of the organism as *F. tularensis*. This could result in a false biosafety alarm, as *F. tularensis* is a Risk Group 3 pathogen and a potential biological weapon [2]. Subsequent failure of prompt treatment may result in poor prognosis with mortality among infected patients. This is especially so for patients with underlying end-stage renal disease and diabetes mellitus, since the latter condition is a strong risk factor for the former [3]. Additionally, having diabetes could alter the immune response patterns to intracellular bacterial infection (e.g., by *Francisella* spp.) in humans [4]. The present report describes a case of *F. philomiragia* bacteraemia reported in Malaysia in a patient with underlying end-stage renal disease and diabetes mellitus.

## Case presentation

A 60 year old male, chronic smoker with underlying diabetes mellitus, hypertension and chronic kidney disease was admitted to Hospital Melaka, Malaysia. He was presented with sudden onset of shortness of breath for a one-day duration associated with failure symptoms of orthopnoea, paroxysmal nocturnal dyspnoea and bilateral lower limb swelling for two months prior to admission. He was compliant to the restriction of fluid of 500 cc/day and denied any past medical history of ischemic heart disease or bronchial asthma. There was no chest pain, palpitations, or profuse sweating. Furthermore, he denied having fever, cough or other symptoms suggestive of infection from home. The patient denied a history of recent travel, exposure to wild animals or recreational water sources or contact with ill patients.

Initial physical examination revealed a sallow appearance; he was tachypnoeic with a respiratory rate of 32 breaths/min, hypertension with blood pressure of 183/120 mm Hg, and hyperglycaemic (GM: 12.3 mmol/L). Auscultation of the lung revealed bi-basal crepitations. Dual rhythm and no murmur were noted upon cardiac examination. Bilateral pedal oedema was palpated up to knee levels. Arterial blood gas taken when the patient was on high flow mask 15 L/min showed type I respiratory failure (pH: 7.31 mm Hg, PCO_2_ 37 mm Hg, PO_2_ 37 mm Hg, HCO_3_: 18.6 mmol/L, SO_2_ of 95%) and hence, he was started on non-invasive ventilation (CPAP PEEP:7.5, FiO_2_:1.0). The patient was treated for Acute Pulmonary Oedema with Non-ST-Elevation Myocardial Infarction (NSTEMI), with underlying chronic kidney disease approaching end stage renal failure (ESRF) with renal anaemia. This was evidenced by the ST depression over lateral leads (I, II, AVL, V_5_ and V_6_) with poor R wave progression, presence of cardiomegaly and lung congestion on a chest radiograph and elevation of troponin I from 1.47 to 2.35. On day two of admission, the patient’s condition deteriorated as shortness of breath worsened after dialysis and crepitations were heard over bilateral lungs up to the upper zone on auscultation. Hence, he was intubated in view of persistent type I respiratory failure and persistent desaturation despite post haemodialysis.

Laboratory tests revealed a white blood cell count of 22,300 cells/mm^3^. There was a predominance of neutrophilia with a differential of 93.4% neutrophils, 1.4% lymphocytes, 3.5% monocytes and 1.5% eosinophils. The C-reactive protein value of 103.5 mg/L indicated the presence of ongoing active bacterial infection and inflammation. Cardiac echocardiogram suggested heart failure with the ejection fraction of 50% with mildly impaired left ventricular systolic function. Neither clots nor pericardial effusion were noted. Repeated chest radiograph post-intubation revealed left upper and right lower zone consolidation suggesting pneumonia. Hence, he was started on intravenous (IV) amoxicillin/clavulanate empirically and the blood culture was sent to the laboratory for further examinations. Subsequently, the empirical antibiotic was escalated to IV piperacillin/tazobactam 2.25 g four times/day to cover for hospital-acquired pneumonia due to a worsening of consolidation over bilateral lung fields on the chest radiograph taken on the third day of admission. However, his condition did not improve with the antibiotic prescribed.

The aerobic blood culture bottle was positive after 48 h of incubation; however, no organism was observed via Gram staining. Greyish, mucoid colonies were observed on blood agar after 24 h of incubation at 37 °C under aerobic condition. Repeated Gram staining of organisms from the aerobic blood culture bottle after re-incubation for 48 h yielded Gram-negative coccobacilli which appeared singly and in pairs, similar to those cultured from blood agar. *F. philomiragia* was identified using the matrix-assisted laser desorption/ionization-time of flight (MALDI-TOF) mass spectrometry (Score value: 2.16) and confirmed by 16S rDNA sequencing [5] (Accession no. LR999753). MALDI-TOF mass spectrometry was performed in-house using the MALDI Microflex LT system (Bruker Daltonik GmbH, Bremen, Germany). The 16S rDNA gene was amplified with three pairs of overlapping primers, sequenced in both directions, aligned and compared with other sequences in the Basic Local Alignment Search Tool (BLAST) database [5]. The patient was started on oral tablet doxycycline 1 mg twice daily for *Fransicella* bacteraemia on day six of admission. His condition improved after commencement of doxycycline and IV furosemide. Additionally, the fluid overload picture and consolidations on a chest radiograph improved as well. He was discharged well on day 26 of admission, after completing one week of piperacillin/tazobactam (to cover empirically for hospital acquired infection) and two weeks of doxycycline.

## Discussion and conclusions

*F. philomiragia* is an intracellular bacterium, belonging to the *Gammaproteobacteria* class and *Francisellaceae* family, which also include other human pathogens such as *F*. *tularensis and Francisella hispaniensis*, the fish pathogens *Francisella noatunensis* subsp. *orientalis* and *Francisella halioticida*, and environmental isolates of *Francisella guangzhouensis* [6]. The organism was previously known as *Yersinia philomiragia* but was reclassified due to the similarities in the unique fatty acid and DNA profiles as the *Franciscella* genus. *F. philomiragia* has some distinctive biochemical features and a DNA hybridization pattern that differentiate it from *F. tularensis* even though both appear as tiny non-motile, strictly aerobic Gram-negative coccobacilli [7]. In contrast to the select agent organism, *F. tularensis* which causes infection in otherwise healthy people [2], *F. philomiragia* has relatively low pathogenicity and is a rare cause of opportunistic infection in immunocompromised patients. Since the aquatic environment (particularly brackish water and saltwater) is a natural habitat for the organism, it is an important etiological medium of francisellosis in wild and farmed fish [1]. Furthermore, it could also infect aquatic amoebae as demonstrated in laboratory experiments [8]. The incidence or prevalence of human *F. philomiragia* infections is not known as health authorities are not required to be notified [1].

Previous studies have revealed that chronic granulomatous disease, hematogenous malignancies (typically myeloproliferative neoplasms), renal-transplantation and near-drowning accidents in saltwater are the recognized risk factors for *F. philomiragia* infection [2, 9–12]. To date, the pathogenesis of *F. philomiragia* is not well understood compared to *F. tularensis*. It has been suggested that inhalation and/or ingestion of contaminated water may increase the risk of infection in immunocompromised patients [1]. Moreover, *F. philomiragia* infection can be acquired through direct cutaneous inoculation. It is capable of infecting reticuloendothelial tissue as seen in a child with chronic granulomatous disease who had crab-induced abrasion and subsequently developed adenitis and pulmonary nodules [2]. The patient in the present case had underlying diabetes mellitus and chronic kidney disease which caused immunosuppression by defects in phagocytosis and microbial killing [13, 14] that might have predisposed him to *F. philomiragia* bacteraemia. However, the patient did not have any history of recent exposure to water activities nor trauma. Besides, he had no history of contact with a contaminated environment such as an air-conditioning cooling tower that might have led to the inhalation of *F. philomiragia*-contaminated aerosols. As such, the source of infection for this patient could not be ascertained. This is similar to the case described by Relich et al. in which *F. philomiragia*-associated pneumonia was reported in a post renal-transplant patient. The patient was receiving immunosuppressant concurrent with acute-on-chronic kidney disease. He had not been exposed to contaminated water sources or a near-drowning event [1]. The patient could also have been inoculated with *F. philomiragia* through the bite of an infected tick. Although transmission of *F. philomiragia* through tick bites has not been reported, *Francisella* DNA sequences have previously been detected in Malaysian ticks [15].

*F. philomiragia* infection can manifest as pneumonia, empyema, vesicular skin lesions, sepsis, splenic microabcesses, peritonitis, or meningitis. Only few cases have been reported globally and the organism has been isolated from the blood, tissues and cerebrospinal fluid [6]. As for this patient, he was initially presented with fluid overload symptoms and then developed pneumonia after two days of admission. This was supported by the presence of consolidations on a chest radiograph, a high level of white blood cells as well as an elevated level of C-reactive protein. The patient exhibited neutrophilia. While neutrophils respond early to *Francisella* infection, excessive neutrophil recruitment could also exacerbate *Francisella*-associated pathogenesis [16]. *F. philomiragia* is likely the causative agent for pneumonia in this patient. No further examination such as the computed tomography scan of the lungs had been performed to identify other possible cause of pneumonia.

*Francisella philomiragia* is an intracellular bacterium of macrophages which is faintly stained on Gram stain as tiny, highly pleomorphic, Gram-negative coccobacillus with bizarre forms on primary isolation. This might explain the reason for no organism being detected on the initial Gram stain of the sample from the positive aerobic blood culture bottle for this patient. As a close relative to *Francisella tularensis,* both appear as smooth, raised, mucoid colonies on chocolate or cysteine-supplemented agar, and have weakly positive catalase tests. However, *F. philomiragia* is cysteine-independent and yields white mucoid colonies in contrast to *F. tularensis* which usually yields greyish mucoid colonies, and is a fastidious organism. Moreover, *F. philomiragia* can be distinguished from *F. tularensis* by a positive Kovaç’s oxidase test, hydrogen sulphide production in triple sugar iron sugar, and delayed gelatine hydrolysis [2, 11]. In this case, the organism was cultured as whitish, mucoid colonies on blood agar after 24 h of incubation under aerobic condition, but no growth was observed on chocolate and MacConkey agars.

Currently, there is no standardized guideline on the susceptibility testing of *Francisella* species and thus, the laboratory data should be interpreted with caution. Although the organism appeared to be susceptible to cephalosporin in vitro, treatment failure involving *F. philomiragia* infection has been recognized [17]. This was further demonstrated in a study by Wenger el al., in which all *F. philomiragia* isolates were beta -lactamase producers and resistant to ampicillin. *F. philomiragia* resistance to cefazolin and cefotaxime has been reported as well [7]. In the present case, the *F. philomiragia* isolate was susceptible to both ciprofloxacin and doxycycline, with the minimum inhibitory concentrations determined for the antibiotics as 0.008 and 1.0 mcg/ml, respectively. The patient responded well to the administered doxycycline and piperacillin/tazobactam antibiotics, an outcome similar to the findings by Relich et al. [1], as evidenced by resolution of the pneumonic patch on a chest radiograph and lung findings on clinical examination.

In conclusion, *F. philomiragia* infection may occur in immunocompromised patients such as the diabetic patient in this study. Clinical suspicion should be raised if patients with known risk factors are presented with pneumonia or pulmonary nodules, these being the most common manifestations of *F. philomiragia* infection. Early diagnosis via accurate laboratory identification of the organism through MALDI-TOF mass spectrometry and molecular technique such as 16S rDNA sequencing are vital for prompt treatment to obtain better outcomes for the afflicted patients.

## Data Availability

Not applicable.

## Abbreviations

ESRF: End stage renal failure
NSTEMI: Non-ST-Elevation Myocardial Infarction
MALDI-TOF: Matrix-assisted laser desorption/ionization-time of flight
16S rDNA: 16S Ribosomal DNA
PCO_2_: Partial Pressure of Carbon Dioxide
PO_2_: Partial Pressure of Oxygen
HCO_3_: Bicarbonate
SO_2_: Oxygen Saturation
CPAP: Continuous Positive Airway Pressure Therapy
PEEP: Positive End-Expiratory Pressure
FiO_2_: Fraction of Inspired Oxygen
IV: Intravenous
BLAST: Basic Local Alignment Search Tool
